# Continuum of maternal health care utilisation in Sub-Saharan African countries: A positive deviance approach

**DOI:** 10.1371/journal.pone.0314779

**Published:** 2025-06-25

**Authors:** Samrawit Mihret Fetene, Melaku Birhanu Alemu, Elsa Awoke Fentie, Tsegaye G. Haile

**Affiliations:** 1 Department of Health Systems and Policy, Institute of Public Health, College of Medicine and Health Sciences, University of Gondar, Ethiopia; 2 Surveillance and Evaluation Research Program, Kirby Institute, University of New South Wales, Sydney, New South Wales, Australia; 3 Curtin School of Population Health, Curtin University, Perth, WA, Australia; 4 Department of Reproductive Health, Institute of Public Health, College of Medicine and Health Sciences, University of Gondar, Gondar, Ethiopia; 5 School of Population Health, Facility of Medicine and Health Science, University of New South Wales, Sydney, Australia; National Research Centre, EGYPT

## Abstract

**Introduction:**

Maternal health is a global priority for achieving Sustainable Development Goal 3.1. However, many mothers in Africa still lack access to the full continuum of care for maternal health (continuum of care). The lowest coverage is often observed among underserved mothers, particularly those who are uneducated and from poor households. Despite these disadvantages, some mothers in every community find effective ways to access a better continuum of care – these are known as positive deviants. However, there is limited evidence to support this concept. Therefore, this study examined the determinants of continuum of health care utilisation among underserved mothers in sub-Saharan African countries.

**Methods:**

Data from the Demographic and Health Surveys of 15 Sub-Saharan African countries with high maternal mortality ratio were used. A positive deviance approach was applied to identify 32,778 underserved mothers using a two-stage stratified sampling technique for the final analysis. A multilevel mixed-effect binary logistic regression analysis was conducted to identify factors associated with being a positive deviant. Finally, an adjusted odds ratio (AOR) with a 95% confidence interval (CI) was used to declare statistically significant determinants.

**Results:**

The overall continuum of care utilisation among underserved mothers was 13.8% (95% CI: 13.5–14.2%). Underserved mothers who were employed (AOR = 1.2; 95% CI: 1.1–1.3), those who had educated husband (AOR = 1.3; 95% CI:1.2–1.4), had one to three children (AOR = 1.2; 95%CI: 1.1–1.3), had a history of pregnancy termination (AOR = 1.3; 95%CI: 1.1–1.4), had the healthcare decision making autonomy (AOR = 1.2; 95% CI: 1.1–1.3), and faced no barriers to accessing health services (AOR = 1.2; 95% CI: 1.0–1.2) were significantly associated with being a positive deviant.

**Conclusion:**

Despite socioeconomic disadvantage, a subset of underserved mothers in sub-Saharan Africa successfully utilised the full continuum of maternal healthcare. Key enabling factors included maternal employment, partner education, smaller family size, prior pregnancy termination, autonomy in healthcare decision-making, and absence of access barriers. These findings support the positive deviance approach as a valuable lens for identifying actionable pathways to improve maternal health coverage. Interventions that amplify these enabling factors could help close equity gaps and accelerate progress toward maternal health targets in high-burden settings.

## Introduction

Maternal health is a global public health priority and a key indicator of community health and development [1]. The World Health Organization (WHO) states that improving maternal and child health is essential for achieving the Sustainable Development Goals [2]. Despite a 34% decline in the global Maternal Mortality Ratio (MMR) from 2000 to 2020, maternal deaths remain alarmingly high [3]. The majority of maternal deaths (94%) occurred in low-resource settings, with sub-Saharan Africa and Southern Asia accounting for about 86% of global maternal deaths in 2017 [4]. This high MMR is linked to low utilisation of antenatal care, limited access to postnatal care, and a lack of skilled birth attendants [5].

According to the WHO, improving the quality, accessibility, and affordability of maternal health services is crucial for reducing the risk of death among mothers [6]. Providing these services as part of a continuum of care approach can significantly lower preventable maternal death, particularly in sub-Saharan Africa [7]. This approach ensures high-impact interventions are delivered throughout pregnancy, childbirth, and the postpartum period [8], leading to 15% reduction in combined maternal and perinatal mortality [9]. However, high dropout rates from this care model, contribute to the high MMR in sub-Saharan Africa, where 86% of women discontinuing care [10].

Many empirical evidences have shown that a mother’s education [11–15] and household wealth status [16–20] are significantly associated with the utilisation of continuum of care, suggesting that improving these factors can enhance service uptake. However, a large number of women in Africa face challenges in accessing continuum of care, particularly those with no formal education and from low-income families [21]. Contributing factors include limited awareness of maternal health services, economic barriers, long distances to health facilities, inadequate transportation, and restricted autonomy in household decision-making [22].

Despite facing significant challenges such as poverty and lack of education, some mothers demonstrate better utilisation of the continuum of care and are referred to as positive deviants. However, there is limited evidence on the determinants associated with being a positive deviant, as the existing literature primarily focuses on the overall population [10,13,15,23–26]. This study uniquely applies a positive deviance approach to identify determinants of full continuum of care utilisation among underserved mothers, focusing on what contributes to being a positive deviant. By examining uncommon but positive behaviours within this population, the study offers practical and scalable strategies for healthcare providers, policymakers, and other stakeholders to enhance care in underserved communities. Therefore, this study aimed to identify the determinants of being a positive deviant in utilisation of continuum of care in sub-Saharan African countries with high maternal mortality ratio, using positive deviance approach.

## Methods

### Data sources and context

We used the Demographic and Health Surveys (DHS) data from 15 sub-Saharan African countries (Benin, Burundi, Cameroon, Chad, Ethiopia, Gambia, Guinea, Liberia, Mali, Mauritania, Nigeria, Sierra Leone, Tanzania, Uganda and Zimbabwe) which were collected using a community-based cross-sectional study design. The DHS is a nationally representative household survey in more than 85 countries worldwide [27].

The countries included in the analysis were selected based on their high MMR in Africa and the availability of recent DHS, as shown in Table 1. We used the WHO thresholds to categorise MMR (maternal deaths per 100,000 live births), defining them as follows: < 100 (very low), 100–299 (low), 300–499 (high), 500–999 (very high), and >1000 (extremely high) [29]. In this study, 15 countries were selected based on their high MMR (≥300 per 100,000 live births) and availability of recent DHS data post-2015, ensuring comparability and relevance to post-Millennium Development Goal assessments. Countries with high MMR but lacking recent DHS data were excluded from the analysis.

**Table 1:** Sub-Saharan African countries with high maternal mortality ratio included in the analysis and their corresponding DHS Year.

Country	Maternal mortality ratio/ 100,000 live births [28]	Recent DHS Year
Chad	1140	2014−15
Sierra Leone	1120	2019
Nigeria	917	2018
Mauritania	766	2021
Liberia	661	2019−20
Gambia	597	2019−20
Guinea	576	2018
Mali	562	2018
Burundi	548	2016−17
Cameroon	529	2018
Tanzania	524	2015−16
Zimbabwe	458	2015
Ethiopia	401	2016
Benin	397	2017−18
Uganda	375	2016

### Sampling procedures and sample size

The DHS employed a two-stage stratified sampling techniques to select the study participants. In the first stage, enumeration areas or clusters were systematically selected, serving as the primary sampling units (PSUs). The selection of PSUs is generally carried out using a sampling frame based on the latest census or other demographic data sources. A subset of clusters is then chosen through probability proportional to size sampling. In the second stage, households were randomly selected for interviews within each selected Enumeration area.

**Fig 1:**
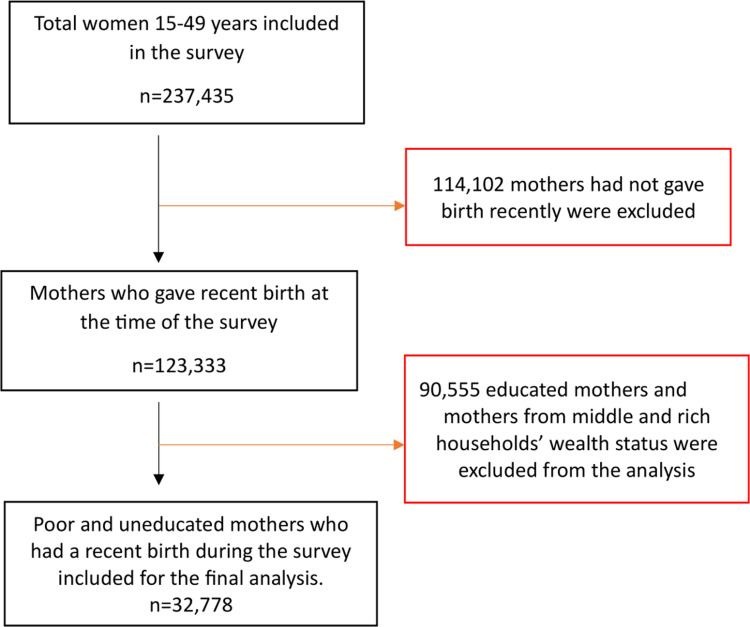
Selection of underserved mothers in 15 sub-Saharan African countries (n = 32,778).

We used individual record data sets (IR file) for this study that consisted of information from all eligible women aged 15–49 years. The source population for this study included all women who had given birth within the five years prior to the survey. Finally, from a total of 237,435 identified women, 32,778 mothers were included for this analysis as described in Fig 1.

### Identification of positive deviant mothers

Anderson’s behavioral model of health service [30] along with most empirical evidence, [11,15,16,18,20,24,31,32] indicates that underserved mothers such as those living in poverty, in rural areas, and with limited education are at risk for low utilisation of maternal health care services. In our analysis, we excluded educated mothers (those who attended primary, secondary, or higher education) and those from middle and rich households, focusing solely on uneducated mothers and those from poor households. We categorised these mothers as “underserved mothers” due to their very low likelihood of continuum of care utilisation, as education and household wealth are key predicators.

Despite being poor and uneducated, some mothers in every community find effective ways to access a better continuum of care, these are known as positive deviants. In this study, positive deviants are defined as poor, uneducated mothers who fully utilised continuum of care. Therefore, the positive deviance approach allows us to observe that at-risk mothers can adopt uncommon but beneficial practices, leading to better outcomes than others with similar risks [33]. This means that, while it is uncommon to find a better continuum of care utilisation among underserved mothers [13,20]; some of them may still achieve a better continuum of care utilisation compared to other underserved mothers.

### Measurement of variables

The outcome variable in this study was the continuum of maternal health care utilisation, which defined as the use of all three key maternal health services: having four and more antenatal care visits, delivering at health facility, and receiving postnatal care within two days after childbirth, all provided by skilled health professionals [23,34,35]. If a mother accessed all three services components, we categorised as having “utilised”, otherwise categorised as “not utilised”. We used ‘continuum of care’ to refer to ‘continuum of care for maternal health’ throughout the document.

Our study assessed independent variables by considering the individual and community-level variables (Table 2) [25,36–39].

**Table 2:** Variable measurements in the study of continuum of care utilisation.

Individual level variables	Measurement
Age of the mother	15-24 years = “0”, 25–34 years = “1” and 35 and above= “2”
Current marital status	Unmarried = “0” and married = “1”. The “unmarried” category encompassed respondents who were single, widowed, divorced, or separated.
Sex of household head	Male = “0” and Female=”1”
Employment status of mother and husband	Unemployed = “0” and employed = “1”. The “employed” category included respondents working in professional, agricultural, skilled and unskilled manual, sales, and clerical occupations.
Educational status of husband	Uneducated = “0 “ and educated = 1 “. The educated category encompassed those who had primary, secondary and higher education.
Mother’s media exposure	It was assessed using three variables: listening to the radio, watching television, and reading a newspaper. It was labeled as “yes = 1” if a woman had exposure to any of the three media sources at least once a week, and “no = 0” if a woman had no exposure to any of them.
Relationship with household head	Head = “0”, wife = “1”, daughter = “2” and others = “3”. The “others” category includes grand-daughter, mother, sister, and co-spouse.
Health Insurance coverage	The respondents asked whether they are covered by health insurance and their response re-coded as: no = “0” and yes = “1”.
Healthcare decision making autonomy	We re-coded women who made healthcare decisions alone or with their partner as “1”, while respondents whose partners made decisions alone for healthcare were re-coded as “0”. Here, “0” represents women with no autonomy in healthcare decision-making, while “1” represents women with autonomy in healthcare decision-making.
Parity	One to three = “0” and four and above = “1”.
History of pregnancy termination	No = “0” and yes = “1”.
Pregnancy Wantedness	No more = “0”, later = “1”, and then = “2”. When we refer to “No more” indicates that the pregnancy of last child was entirely unwanted; “Later” indicates that women did not desire the pregnancy of last child initially but later accepted it over time and “Then”, it means that women desired the pregnancy of last child at the time of conception.
Informed about potential pregnancy complications	No = “0” and yes = “1”
**Community level variables**	**Measurements**
Community level media exposure	A community-level media exposure is measured by the proportion of mother who have been exposed to at least one media and categorised based on the median value as “low=0” (communities with <50% of mother exposed) and “high=1” (communities with ≥50% of mother exposed) since proportion of media exposure level among mother was not normally distributed.
Difficulty of getting to the nearest health facility	Mothers were asked if the distance to the health facility a big problem for them was, and their responses were recoded as follows: “big problem = 0” and “not big problem= 1.”

### Data processing and analysis

The data were extracted, cleaned, re-coded, and analysed using STATA version 16 statistical software. Descriptive statistics were presented through tables, figures, and narrative descriptions. We performed a weighted data analysis using individual sample weights assigned to women to account for the complexities of the DHS multi-stage cluster sampling design. Additionally, we considered clustering and stratification in our analyses by using the “svyset” command in Stata [26].

The DHS data were collected using multistage stratified cluster sampling techniques; resulting in a hierarchical structure where individuals are nested within communities. Mothers selected and interviewed within the same cluster are likely to share more similarities than those from different clusters. This indicates a need to account for variability between clusters by using advanced analytical models. Therefore, we employed a multilevel analysis method to identify determinants of being positive deviant.

The Interclass Correlation Coefficient (ICC) was used to evaluate significant variation in group clustering. In our study, we found an ICC of 0.075, indicating significant variation in utilisation of continuum of care among underserved mothers across different clusters. To account for this clustering effect, we fitted three models: Model I (which includes no explanatory variables), Model II (which includes individual-level factors), and Model III (which account for both individual and community-level factors), for accounting the clustering effect.

The model comparison and finesses were checked using ICC, Akaike’s Information Criterion (AIC), and deviance (−2* log-likelihood ratio). A model (model III) with the lowest AIC and deviance was selected. Multicollinearity was tested using the Generalized variance inflation factor (GVIF) [40]. We found that the GVIF values for all predictor variables included in the final model were less than 1.5, indicating that there was no significant multicollinearity. Then we used multilevel fixed effect binary logistic regression to account for clustering effect and identify determinants of being positive deviant. Finally, after adjusting for both the individual- and community-level factors, we used adjusted odds ratios (AOR) with 95% confidence interval (CI) and a p-value of less than 0.05 to identify statistically significant determinants of being positive deviant.

### Missing data handling

A guide to DHS statistics [41] was used to ensure data quality and handle the missing data. Accordingly, participants with missing values or “do not know” responses to place of delivery (23) were considered as “home delivery”, and to antenatal care visits (78) were considered as “less than four antenatal care visits”. Furthermore, when the timing of the PNC check was reported as in days, the missing or “don’t know” (137) was considered as “no PNC check” in the first two days. In total, 238 data items (0.73% of the dataset) were imputed following the guidelines from the DHS statistics manual.

### Ethical considerations

We submitted a formal request to access DHS data for sub-Saharan African countries on 2 February 2023, and permission was granted on 6 February 2023. Participants provided informed consent before participating in the surveys. There are no names of individuals or household addresses/ personal identifiers in the data files.

## Results

### Socio-demographic and economic characteristics

The socio-demographic and economic characteristics of respondents included in this analysis are presented in Table 3. The mean age of respondents was 30 ± 7.5 years, 25.9% were employed, and nearly 15% of them were household heads. Furthermore, 93.6% of respondents are married, and of these married mothers, 77.8% of their husbands are uneducated.

**Table 3:** Socio-demographic related characteristics of respondents (n = 32,778).

Variables	Frequency (n)	Percentage (%)
Age of the mothers (in years)		
15-24	7,647	23.3
25-34	14,697	44.9
35 and above	10,434	31.8
Current marital status		
Unmarried	1,569	4.8
Married	31,209	95.2
Sex of household head		
Male	27,863	85.1
Female	4,915	14.9
Employment status of mother		
Unemployed	8,505	25.9
Employed	24,273	74.1
Employment status of husband		
Unemployed	2650	8.6
Employed	28,240	91.4
Educational status of husband		
Uneducated	24,049	77.8
Educated	6841	22.2
Mother’s media exposure		
No	23,503	71.7
Yes	9,275	28.3
Relationship with household head		
Head	3,573	10.8
Wife	24,151	73.7
Daughter	3,066	9.4
Others^*^	1,988	6.1
Health insurance coverage		
No	30,136	91.9
Yes	2,642	8.1
Healthcare decision making autonomy		
No	20,554	62.7
Yes	12,224	37.3

*Others*

* *=granddaughter, mother, sister, co-spouse*

### Obstetric-related characteristics of respondents

Of the total mothers, 12.6% had a history of pregnancy termination. Nearly half (51.9%) of the respondents had four or more children as presented in Table 4. Furthermore, only 3% of respondents were informed about potential pregnancy complications.

**Table 4:** Obstetric-related characteristics of respondents (n = 32,778).

Variables	Frequency (n)	Percentage (%)
Parity		
One – Three	17,026	51.9
Four and above	15,752	48.1
History of pregnancy termination		
No	28,644	87.4
Yes	4,134	12.6
Pregnancy Wantedness		
No more	1,730	5.3
Later	3,377	10.3
Then	27,671	84.4
Informed about potential pregnancy complications		
No	31,751	96.9
Yes	1,027	3.1

### Community-level characteristics

The community-level characteristics for continuum of care utilisation are presented in Table 5. Most respondents (91.6%) were rural dwellers, and 54% lived closer to health facilities.

**Table 5:** Community-level characteristics for continuum of care utilisation (n = 32,778).

Variables	Frequency (n)	Percentage (%)
Residence		
Urban	2,740	8.4
Rural	30,038	91.6
Difficulty of getting to the nearest health facility		
Big problem	16,500	53.8
Not big problem	14,168	46.2
Community-level media exposure		
Low	16,366	49.9
High	16,412	50.1

### Proportion of continuum of care utilisation

**Fig 2:**
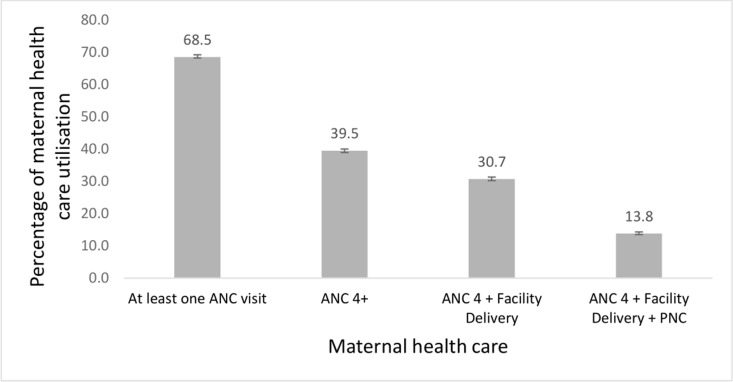
Continuum of maternal health care utilisation among underserved mothers (n = 32,778).

**Fig 3:**
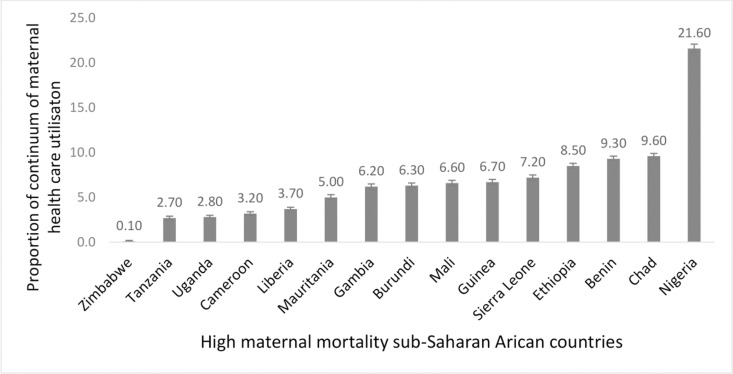
Proportion of continuum of maternal care service utilisation among underserved mothers by countries (n = 32,778).

A total of 12,954 mothers (39.5%, 95% CI: 38.9–40.0) had four and above antenatal care visits during their most recent pregnancy. In addition, 21,761 mothers (66%, 95% CI: 65.9–66.9) delivered their babies in the health facilities. We found that 72.8% mothers (95%CI: 72.3–73.3) received a postnatal care visit within the first two days after giving birth. Among mothers who had their first antenatal care visit, only 39.5% attended four or more visits. Of those who had four or more antenatal care visits, only 30.7% gave birth in a health facility. Overall, 13.8% (95% CI: 13.5–14.3) of underserved mothers utilised the full continuum of care (Fig 2), with country-level variations from 0.1% (95% CI: 0.08–0.2) in Zimbabwe to 21.6% (95% CI: 21.2–22.1) in Nigeria, as shown in Fig 3.

### Determinants of being a positive deviant

#### Random effect estimates.

The random effect estimates revealed that 7.5% (ICC = 0.075 95% CI:6.2,9.0) of the variation in the utilisation of continuum of care among underserved mothers can be attributed to clustering (Table 6). As a result, we employed fixed effect estimates to identify the determinants of being a positive deviant.

**Table 6:** Random-intercept model of multilevel analysis of continuum of care utilisation (n = 32,778).

Measure of variations	Model I	Model II	Model III
LLR	−12,732.1	−11,748.4	−11,057.3
ICC	7.5	7.2	7.1
Deviance (−2*LLR)	25,464.2	23,496.8	22,114.6
AIC	25,485.0	23,514.9	22,138.6

LLR = Log Likelihood Ratio, ICC = Intraclass Correlation Coefficient, AIC = Akaike’s Information Criterion

*Model I = a model without independent variables, Model II = a model with individual-level variables, Model III = a model adjusted for both the individual and community-level variables*

#### Fixed effect estimates.

After adjusting for individual- and community-level factors, several factors were found to be significantly associated with being a positive deviant. These include the mother’s employment status, her husband’s educational level, history of pregnancy termination, parity, her autonomy in making healthcare decisions, and the distance to the nearest health facility (Table 7).

**Table 7:** Determinants of being a positive deviant in continuum of care utilisation (n = 32,778).

Variables	Continuum of care		COR (95%CI)	Model II AOR (95% CI)	Model III AOR (95% CI)
	Yes (Positive deviant) n (%)	No (Not positive deviant) n (%)			
Employment status of mother					
Unemployed	927 (10.9)	7,578 (89.1)	1	1	1
Employed	3,621 (14.9)	20,652 (85.1)	1.3 (1.2–1.4)	1.3 (1.1–1.4)	1.2 (1.1–1.3)^*^
Sex of household head					
Male	3,808 (13.7)	24,055 (86.3)	1	1	1
Female	740 (15.1)	4,175 (84.9)	1.1 (1.0–1.2)	1.0 (0.9–1.1)	1.0 (0.9–1.1)
Educational status of husband					
Uneducated	3,000 (12.5)	21,049 (87.5)	1	1	1
Educated	1,190 (17.4)	5,651 (82.6)	1.4 (1.3–1.5)	1.3 (1.2–1.4)	1.3 (1.2–1.4)^*^
Employment status of husband					
Unemployed	317 (11.9)	2,342 (88.1)	1	1	1
Employed	4,231 (14.1)	25,888 (85.9)	1.2 (1.0–1.3)	1.1 (0.9–1.3)	1.1 (0.9–1.3)
History of pregnancy termination					
No	3,834 (13.4)	24,810 (86.6)	1	1	1
Yes	714 (17.3)	3,420 (82.7)	1.3 (1.2–1.4)	1.3 (1.2–1.4)	1.3 (1.1–1.4)^*^
Parity					
One – Three	2,452 (14.4)	14,574 (85.6)	1.1 (1.0–1.2)	1.2 (1.1–1.3)	1.2 (1.1–1.3)^*^
Four and above	2,096 (13.3)	13,656 (86.7)	1	1	1
Healthcare decision making autonomy					
No	1,879 (15.4)	10,345 (84.6)	1.2 (1.1–1.3)	1.3 (1.2–1.4)	1.2 (1.1–1.3)^*^
Yes	2,669 (12.9)	17,885 (87.1)	1	1	1
Residence					
Urban	372 (13.6)	2,368 (86.4)	1.0 (0.9–1.2)		0.9 (0.8–1.1)
Rural	4,176 (13.9)	25,862 (86.1)	1		1
Difficulty of getting to the nearest health facility					
Big problem	2,191 (13.28)	14,309 (86.7)	1		1
Not big problem	2,177 (15.4)	11,991 (84.6)	1.1 (1.0–1.2)		1.1 (1.0–1.2)^*^
Community level media exposure					
Low	2,246 (13.7)	14,120 (86.3)	1		1
High	2,302 (14.1)	14,110 (85.9)	1.0 (0.9–1.1)		1.0 (0.9–1.12)

*AOR: Adjusted Odds Ratio, COR: Crude Odds Ratio*

*Model II: Adjusted for individual-level factors, Model III: Adjusted for both individual and community-level factors (full model)*

* *Statistically significant at P-value < 0.05 in the full model*

The odds of being a positive deviant in the utilisation of the continuum of care were 1.2 times higher among employed mothers compared to unemployed mothers (AOR = 1.2; 95%CI: 1.1–1.3). Mothers whose husbands were educated had 1.3 times higher odds of being positive deviants in the utilisation of the continuum of care compared to their counterparts (AOR = 1.3; 95%CI: 1.2–1.4). The odds of being a positive deviant in the utilisation of the continuum of care were 1.2 times higher among mothers with one to three children compared to those with four or more children (AOR = 1.2; 95%CI: 1.1–1.3). Mothers with a history of pregnancy termination had 1.3 times higher odds of being positive deviants in the utilisation of the continuum of care compared to their counterparts (AOR = 1.3; 95%CI: 1.1–1.4). The odds of being a positive deviant in the utilisation of the continuum of care were 1.2 times higher among mothers with healthcare decision-making autonomy compared to those without it (AOR = 1.2; 95% CI:1.1–1.3). The odds of being a positive deviant in the utilisation of the continuum of care were 1.1 times higher among mothers who did not have a big problem in getting to health facility compared to their counterparts (AOR = 1.1; 95%CI: 1.0–1.2).

## Discussion

Our positive deviance approach highlighted that some underserved mothers were able to fully utilise the continuum of maternal care, despite facing considerable social and economic challenges. We found that 13.8% (95% CI: 13.5–14.25) of these mothers achieved fully utilisation of care. Notably, certain behavioural and contextual factors were significantly associated with positive deviance. These findings underscore the potential to identify and amplify enabling factors that allow some women to overcome barriers to care in resource-constrained settings.

Our results are lower than previous studies in sub-Saharan Africa (35.81%) [13], South Asia (24.5%) [10], and Southeast Asia (21.9%) [42]. Differences in results could stem from variations in health care infrastructure, cultural norms, and measurement methodologies across studies. For example, earlier studies looked at the general population, while our study focused specifically on poor and uneducated mothers. Mothers without formal education may not be aware of the benefits of continuum of care. In addition, poor and uneducated mothers are often less engaged in paid work, more financially dependent, and typically have less decision-making power regarding their health. This can hinder their ability to achieve better health outcomes [43]. Even though many countries offer full or partial exemptions for maternal health services, underserved mothers still face challenges due to indirect costs associated with accessing continuum of care. Therefore, it is essential to pay special attention to these underserved mothers to improve their use of continuum of care.

We found that the odds of being a positive deviant in the utilisation of the continuum of care were higher among employed mothers compared to unemployed mothers. This aligns with previous studies conducted in Ethiopia [23,34,36], and Egypt [35], though they did not specifically apply a positive deviance approach. The employed mothers may enjoy economic independence, giving them greater autonomy to make decisions about their healthcare compared to unemployed mothers [36]. This suggests that even if mothers are poor and uneducated, their use of continuum of care improves when they are financially independent. Therefore, the government should create opportunities for mothers to work and achieve financial independence.

Mothers whose husbands were educated had higher odds of being positive deviant in utilisation of continuum of care compared to their counterparts. This finding is consistent with studies conducted in Egypt [35], Gambia [44], North India [25], Pakistan [39], sub-Saharan African countries [7] and Southeast Asia [42]. While these studies include mothers from various educational and economic backgrounds, having an educated husband can also benefit poor and uneducated mothers. Educated husbands often communicate better with their wives, are more motivated to discuss the advantages of utilising continuum of care, and tend to grant their wives greater independence [35].

This study found that mothers with one to three children were more likely positive deviant in utilisation of continuum of care compared to those with four or more children. Despite differences in study design and focus, this finding was supported by studies done in Ethiopia [14], Pakistan [39] and South Asia and sub-Saharan African countries [10]. This could be due to the fact that mothers with fewer children are more sensitive to pregnancy-related complications and may be more eager to utilise the continuum of care [45] whereas mothers with higher parity have difficulty accessing services due to childcare responsibilities and resource constraints [46]. Moreover, these mothers may also rely on previous pregnancy experiences [47]. On the contrary, a study done in Ethiopia revealed that mothers with four or more children had higher odds of continuum of care utilisation than their counterparts [48]. This could be that mothers with high parity might have better information about the advantages of utilising continuum of care, and they may have faced pregnancy-related complications before. As a result, they may take precautions for a subsequent pregnancy by utilising a continuum of care.

Our findings show that mothers with a history of pregnancy termination had higher odds of being positive deviant in utilisation of the continuum of care compared to their counterparts. This is consistent with another study conducted in sub-Saharan African countries [13]. It suggests that an experience of pregnancy termination may encourage these mothers to seek better care for their current pregnancies. They might be more cautious about the outcomes of their current pregnancies due to previous negative experiences [49]. Moreover, support and care from health professionals for mothers with high-risk pregnancies can enhance their willingness to seek health services [13].

The odds of being a positive deviant in the utilisation of the continuum of care were higher among mothers with healthcare decision-making autonomy compared to those without it. This aligns with results from previous studies conducted in Ethiopia [36,48,50,51], Gambia [44], Pakistan [39], sub-Saharan African countries [7], Cambodia [52], Albania [53], and Nepal [54]. This implies that, regardless of a mother’s educational background or economic status, having decision-making power in healthcare can enhance her use of maternal health services. A randomized controlled trial conducted in Egypt revealed that a community-based intervention, which improved awareness of women’s rights to receive care at health units, empowered women during the childbearing period. This, in turn, led to greater autonomy in making decisions to seek optimal prenatal, natal, and postnatal healthcare [55,56]. Therefore, policymakers should prioritise empowering mothers to make their own healthcare decisions, as this could significantly increase the utilisation of continuum of care.

Mothers were more likely to utilise a continuum of care if they did not face major difficulties reaching health facilities. This finding is consistent with studies done in Indonesia [37], Gambia [44], Pakistan [20], and sub-Saharan African countries [26]. It suggests that when access to health facilities is easier, care utilisation improves, even for underserved mothers who may otherwise struggle due to long travel distances. However, distance, along with transportation availability and costs, remains a significant barrier to accessing care [57]. To enhance continuum of care utilisation, governments should focus on increasing the number of health facilities and ensuring maternal health services are easy for all mothers to access.

### Implications of the study

Our findings indicate that the positive deviant behaviors of mothers with challenging circumstances (those who are uneducated and poor) can enhance the continuum of care utilisation. These beneficial behaviors should be integrated into maternal health strategies and encouraged within the broader community. Integrated programs should focus on vocational training for women, male engagement in maternal health education, and community health worker initiatives to enhance continuum of care utilisation. Additionally, the challenge of distance to health facilities suggests that sub-Saharan countries need to increase the number of health facilities and ensure maternal health services are easily accessible to mothers.

### Strengths and limitations

Although many studies have explored the factors influencing maternal healthcare utilisation, our study is unique in applying the positive deviance approach. This approach focuses on identifying factors that enable poor and uneducated mothers to maintain consistent care, despite facing similar challenges as others in their community. This approach identifies context-specific behaviors that require minimal external resources, making them feasible for large-scale implementation in resource-limited settings.

This study offers valuable insights, but it is necessary to consider its limitations. Our study included women who had given birth within the five years prior to the survey. As a result, mothers were expected to recall whether they utilised each component of the continuum of care, the number of ANC visits, whether the services were provided by skilled health professionals and other details. This reliance on self-reported data may lead to underreporting or overreporting of service utilisation, a limitation known as recall bias. This could result a mother might be incorrectly classified as a positive deviant due to overreporting or not recognised as one due to underreporting. However, the DHS Program employed standardised data collection tools and techniques, including data editing and imputation, to help minimise the effect of recall bias.

Even though we included surveys conducted after 2015 to examine the pattern of continuum of care utilisation in the post-MDG era, differences in survey timing may still affect the pooled estimates. This is because countries have experienced different changes in health policies, socio-political priorities, and infrastructure over the years. Such changes may influence accessibility, availability, quality, and ultimately the utilisation of maternal health services, thereby introducing heterogeneity into the pooled analysis. To address this issue, we recommend that future researchers use time-specific or longitudinal data. Furthermore, combining countries with high, very high, and extremely high maternal mortality ratios into a single “high” category may not accurately reflect the unique contexts and challenges faced by countries with extremely high maternal mortality rates. As result we recommend stratified analysis in future research.

## Conclusions

Nearly one in seven mothers are positive deviants in 15 sub-Saharan African countries with high maternal mortality ratio; despite facing socioeconomic challenges these mothers achieve full continuum of care utilisation. Underserved mothers who were employed, had an educated husband, had fewer than four children, possessed healthcare decision-making autonomy, and did not face significant distance issues to the nearest health facility were the positive behaviours that increased their continuum of care utilisation. Policymakers should implement targeted interventions promoting financial independence, male education, and maternal autonomy to improve utilisation of continuum of care among underserved mothers. Achieving SDG 3.1 requires leveraging locally driven solutions, such as those identified in this study, to sustainably reduce maternal mortality in sub-Saharan Africa.

## References

[1] Black RE, Levin C, Walker N, Chou D, Liu L, Temmerman M, et al. Reproductive, maternal, newborn, and child health: key messages from Disease Control Priorities 3rd Edition. Lancet. 2016;388(10061):2811–24. doi: 10.1016/S0140-6736(16)00738-8 27072119

[2] Organization WH. Health in 2015: from MDGs, millennium development goals to SDGs, sustainable development goals. 2015.

[3] World Health Organisation. Maternal Mortality Report. [cited 2025 Mar 10]. Available from: https://www.who.int/news-room/fact-sheets/detail/maternal-mortality.

[4] Organization WH. Trends in maternal mortality 2000 to 2017: estimates by WHO, UNICEF, UNFPA, World Bank Group and the United Nations Population Division: executive summary. In: World Health Organization; 2019.

[5] Aboagye RG, Okyere J, Ahinkorah BO, Seidu A-A, Zegeye B, Amu H, et al. Health insurance coverage and timely antenatal care attendance in sub-Saharan Africa. BMC Health Serv Res. 2022;22(1):181. doi: 10.1186/s12913-022-07601-6 35148769 PMC8840787

[6] Organization WH. Maternal mortality: fact sheet: to improve maternal health, barriers that limit access to quality maternal health services must be identified and addressed at all levels of the health system. 2014.

[7] Seidu A-A, Ahinkorah BO, Aboagye RG, Okyere J, Budu E, Yaya S. Continuum of care for maternal, newborn, and child health in 17 sub-Saharan African countries. BMC Health Serv Res. 2022;22(1):1394. doi: 10.1186/s12913-022-08693-w 36419060 PMC9682703

[8] Tinker A, ten Hoope-Bender P, Azfar S, Bustreo F, Bell R. A continuum of care to save newborn lives. Lancet. 2005;365(9462):822–5. doi: 10.1016/S0140-6736(05)71016-3 15752509

[9] Kikuchi K, Ansah EK, Okawa S, Enuameh Y, Yasuoka J, Nanishi K, et al. Effective linkages of continuum of care for improving neonatal, perinatal, and maternal mortality: a systematic review and meta-analysis. PLoS One. 2015;10(9):e0139288. doi: 10.1371/journal.pone.0139288 26422685 PMC4589290

[10] Singh K, Story WT, Moran AC. Assessing the continuum of care pathway for maternal health in South Asia and Sub-Saharan Africa. Matern Child Health J. 2016;20(2):281–9. doi: 10.1007/s10995-015-1827-6 26511130 PMC4740215

[11] Sserwanja Q, Mukunya D, Nabachenje P, Kemigisa A, Kiondo P, Wandabwa JN, et al. Continuum of care for maternal health in Uganda: a national cross-sectional study. PLoS One. 2022;17(2):e0264190. doi: 10.1371/journal.pone.0264190 35202413 PMC8870527

[12] Sakuma S, Yasuoka J, Phongluxa K, Jimba M. Determinants of continuum of care for maternal, newborn, and child health services in rural Khammouane, Lao PDR. PLoS One. 2019;14(4):e0215635. doi: 10.1371/journal.pone.0215635 31013303 PMC6478320

[13] Hunie Asratie M, Belay DG. Pooled prevalence and determinants of completion of maternity continuum of care in Sub-Saharan Africa: a multi-country analysis of recent demographic and health surveys. Front Glob Womens Health. 2022;3:869552. doi: 10.3389/fgwh.2022.869552 35692945 PMC9174640

[14] Gebremedhin AF, Dawson A, Hayen A. Determinants of continuum of care for maternal, newborn, and child health services in Ethiopia: analysis of the modified composite coverage index using a quantile regression approach. PLoS One. 2023;18(1):e0280629. doi: 10.1371/journal.pone.0280629 36662768 PMC9858465

[15] Sertsewold SG, Debie A, Geberu DM. Continuum of maternal healthcare services utilisation and associated factors among women who gave birth in Siyadebirena Wayu district, Ethiopia: community-based cross-sectional study. BMJ Open. 2021;11(11):e051148. doi: 10.1136/bmjopen-2021-051148 34824117 PMC8627416

[16] Wang W, Hong R. Levels and determinants of continuum of care for maternal and newborn health in Cambodia-evidence from a population-based survey. BMC Pregnancy Childbirth. 2015;15:62. doi: 10.1186/s12884-015-0497-0 25885596 PMC4371879

[17] Mohan D, LeFevre AE, George A, Mpembeni R, Bazant E, Rusibamayila N, et al. Analysis of dropout across the continuum of maternal health care in Tanzania: findings from a cross-sectional household survey. Health Policy Plan. 2017;32(6):791–9. doi: 10.1093/heapol/czx005 28334973

[18] Chalise B, Chalise M, Bista B, Pandey AR, Thapa S. Correlates of continuum of maternal health services among Nepalese women: evidence from Nepal Multiple Indicator Cluster Survey. PLoS One. 2019;14(4):e0215613. doi: 10.1371/journal.pone.0215613 31002686 PMC6474612

[19] Ahmed R, Sultan M, Abose S, Assefa B, Nuramo A, Alemu A, et al. Levels and associated factors of the maternal healthcare continuum in Hadiya zone, Southern Ethiopia: a multilevel analysis. PLoS One. 2022;17(10):e0275752. doi: 10.1371/journal.pone.0275752 36215257 PMC9550044

[20] Gandhi S, Gandhi S, Dash U, Suresh Babu M. Predictors of the utilisation of continuum of maternal health care services in India. BMC Health Serv Res. 2022;22(1):602. doi: 10.1186/s12913-022-07876-9 35513830 PMC9069727

[21] Shabaya J, Konadu‐Agyemang K. Unequal access, unequal participation: some spatial and socio‐economic dimensions of the gender gap in education in Africa with special reference to Ghana, Zimbabwe and Kenya. Compare: J Comp Int Educ. 2004;34(4):395–424. doi: 10.1080/0305792042000294805

[22] Bobo FT, Asante A, Woldie M, Dawson A, Hayen A. Evaluating equity across the continuum of care for maternal health services: analysis of national health surveys from 25 sub-Saharan African countries. Intern. 2023;22(1):239. 10.1186/s12939-023-02047-6 PMC10656898 37978385

[23] Chaka EE, Parsaeian M, Majdzadeh R. Factors associated with the completion of the continuum of care for maternal, newborn, and child health services in Ethiopia. Multilevel model analysis. Int J Prev Med. 2019;10:136. doi: 10.4103/ijpvm.IJPVM_26_19 31516677 PMC6711120

[24] Atnafu A, Kebede A, Misganaw B, Teshome DF, Biks GA, Demissie GD, et al. Determinants of the continuum of maternal healthcare services in Northwest Ethiopia: findings from the primary health care project. J Pregnancy. 2020;2020:4318197. doi: 10.1155/2020/4318197 32908704 PMC7471826

[25] Kushwaha P, Mehnaz S, Ansari MA. Continuum of maternal health care services among peri-urban women- a community-based cross-sectional study in North India. Int J Community Med Public Health. 2022;9(10):3739. doi: 10.18203/2394-6040.ijcmph20222565

[26] Alem AZ, Shitu K, Alamneh TS. Coverage and factors associated with completion of continuum of care for maternal health in sub-Saharan Africa: a multicountry analysis. BMC Pregnancy Childbirth. 2022;22(1):422. doi: 10.1186/s12884-022-04757-1 35590260 PMC9121540

[27] Corsi DJ, Neuman M, Finlay JE, Subramanian SV. Demographic and health surveys: a profile. Int J Epidemiol. 2012;41(6):1602–13. doi: 10.1093/ije/dys184 23148108

[28] World Health Organization. WHO recommendations on postnatal care of the mother and newborn. Geneva: World Health Organization; 2014. Available from: https://apps.who.int/iris/handle/10665/97603 24624481

[29] Trends in maternal mortality 2000 to 2020: estimates by WHO, UNICEF, UNFPA, World Bank Group and UNDESA/Population Division. [cited 2024 Jul 19]. Available from: https://www.who.int/publications/i/item/9789240068759

[30] Andersen RM. Revisiting the behavioral model and access to medical care: does it matter? J Health Soc Behav. 1995;36(1):1–10. 7738325

[31] Sserwanja Q, Musaba MW, Mutisya LM, Olal E, Mukunya D. Continuum of maternity care in Zambia: a national representative survey. BMC Pregnancy Childbirth. 2021;21(1):604. doi: 10.1186/s12884-021-04080-1 34482830 PMC8420052

[32] Fetene SM, Gebremedhin T: Uptake of postnatal care and its determinants in Ethiopia: a positive deviance approach. BMC Pregnancy and Childbirth. 2022, 22(1):601. 35897004 10.1186/s12884-022-04933-3 PMC9327392

[33] Marsh DR, Schroeder DG, Dearden KA, Sternin J, Sternin M. The power of positive deviance. BMJ. 2004;329(7475):1177–9. doi: 10.1136/bmj.329.7475.1177 15539680 PMC527707

[34] Haile D, Kondale M, Andarge E, Tunje A, Fikadu T, Boti N. Level of completion along continuum of care for maternal and newborn health services and factors associated with it among women in Arba Minch Zuria woreda, Gamo zone, Southern Ethiopia: a community based cross-sectional study. PLoS One. 2020;15(6):e0221670. doi: 10.1371/journal.pone.0221670 32511230 PMC7279583

[35] Hamed A, Mohamed E, Sabry M. Egyptian status of continuum of care for maternal, newborn, and child health: Sohag Governorate as an example. Int J Med Sci Public Health. 2018;:1. doi: 10.5455/ijmsph.2018.0102607032018

[36] Addisu D, Mekie M, Melkie A, Abie H, Dagnew E, Bezie M, et al. Continuum of maternal healthcare services utilization and its associated factors in Ethiopia: a systematic review and meta-analysis. Womens Health (Lond). 2022;18. doi: 10.1177/17455057221091732 35412408 PMC9008832

[37] Andriani H, Rachmadani SD, Natasha V, Saptari A. Continuum of care in maternal, newborn and child health in Indonesia: evidence from the Indonesia Demographic and Health Survey. J Public Health Res. 2022;11(4). doi: 10.1177/22799036221127619 36249543 PMC9554135

[38] Hunie Asratie M, Belay DG. Pooled Prevalence and determinants of completion of maternity continuum of care in Sub-Saharan Africa: a multi-country analysis of recent demographic and health surveys. Front Glob Womens Health. 2022;3:869552. doi: 10.3389/fgwh.2022.869552 35692945 PMC9174640

[39] Iqbal S, Maqsood S, Zakar R, Zakar MZ, Fischer F. Continuum of care in maternal, newborn and child health in Pakistan: analysis of trends and determinants from 2006 to 2012. BMC Health Serv Res. 2017;17(1):189. doi: 10.1186/s12913-017-2111-9 28279186 PMC5345258

[40] Hair JF. Multivariate data analysis. 2009.

[41] Croft TN, Marshall AM, Allen CK, Arnold F, Assaf S, Balian S. Guide to DHS statistics. Rockville: ICF; 2018. pp. 645.

[42] Andriani H, Rahmawati ND, Fauzia S, Kosasih RI. Population-based study on the maternal-newborn-child health continuum of care: evidence from lower-middle-income countries in Southeast Asia. Asia Pac J Public Health. 2022;34(5):547–56. doi: 10.1177/10105395221088615 35392673

[43] Kea AZ, Tulloch O, Datiko DG, Theobald S, Kok MC. Exploring barriers to the use of formal maternal health services and priority areas for action in Sidama zone, southern Ethiopia. BMC Pregnancy Childbirth. 2018;18(1):96. doi: 10.1186/s12884-018-1721-5 29649972 PMC5897996

[44] Oh J, Moon J, Choi JW, Kim K. Factors associated with the continuum of care for maternal, newborn and child health in The Gambia: a cross-sectional study using Demographic and Health Survey 2013. BMJ Open. 2020;10(11):e036516. doi: 10.1136/bmjopen-2019-036516 33243786 PMC7692971

[45] Muluneh AG, Kassa GM, Alemayehu GA, Merid MW: High dropout rate from maternity continuum of care after antenatal care booking and its associated factors among reproductive age women in Ethiopia, Evidence from Demographic and Health Survey 2016. PloS One. 2020, 15(6):e0234741. 10.1371/journal.pone.0234741 PMC7292400 32530948

[46] Mathole T, Lindmark G, Majoko F, Ahlberg BM. A qualitative study of women’s perspectives of antenatal care in a rural area of Zimbabwe. Midwifery. 2004;20(2):122–32. doi: 10.1016/j.midw.2003.10.003 15177855

[47] Tarekegn SM, Lieberman LS, Giedraitis V. Determinants of maternal health service utilization in Ethiopia: analysis of the 2011 Ethiopian Demographic and Health Survey. BMC Pregnancy Childbirth. 2014;14:161. doi: 10.1186/1471-2393-14-161 24886529 PMC4022978

[48] Shitie A, Assefa N, Dhressa M, Dilnessa T. Completion and factors associated with maternity continuum of care among mothers who gave birth in the last one year in Enemay District, Northwest Ethiopia. J Pregnancy. 2020;2020:7019676. doi: 10.1155/2020/7019676 32953177 PMC7481927

[49] Suryanarayana R, Chandrappa M, Santhuram AN, Prathima S, Sheela SR. Prospective study on prevalence of anemia of pregnant women and its outcome: a community based study. J Family Med Prim Care. 2017;6(4):739–43. doi: 10.4103/jfmpc.jfmpc_33_17 29564255 PMC5848390

[50] Cherie N, Abdulkerim M, Abegaz Z, Walle Baze G. Maternity continuum of care and its determinants among mothers who gave birth in Legambo district, South Wollo, northeast Ethiopia. Health Sci Rep. 2021;4(4):e409. doi: 10.1002/hsr2.409 34754945 PMC8562404

[51] Asratie MH, Muche AA, Geremew AB. Completion of maternity continuum of care among women in the post-partum period: Magnitude and associated factors in the northwest, Ethiopia. PLoS One. 2020;15(8):e0237980. doi: 10.1371/journal.pone.0237980 32853268 PMC7451525

[52] Chham S, Radovich E, Buffel V, Ir P, Wouters E. Determinants of the continuum of maternal health care in Cambodia: an analysis of the Cambodia demographic health survey 2014. BMC Pregnancy Childbirth. 2021;21(1):410. doi: 10.1186/s12884-021-03890-7 34078318 PMC8170811

[53] Sado L, Spaho A, Hotchkiss DR. The influence of women’s empowerment on maternal health care utilization: evidence from Albania. Soc Sci Med. 2014;114:169–77. doi: 10.1016/j.socscimed.2014.05.047 24929918

[54] Adhikari R. Effect of Women’s autonomy on maternal health service utilization in Nepal: a cross sectional study. BMC Womens Health. 2016;16:26. doi: 10.1186/s12905-016-0305-7 27177683 PMC4867085

[55] Metwally AM, Saleh RM, El-Etreby LA, Salama SI, Aboulghate A, Amer HA, et al. Enhancing the value of women’s reproductive rights through community based interventions in upper Egypt governorates: a randomized interventional study. Int J Equity Health. 2019;18(1):146. doi: 10.1186/s12939-019-1042-y 31533741 PMC6751807

[56] Metwally AM, Abdel-Latif GA, Mohsen A, El Etreby L, Elmosalami DM, Saleh RM, et al. Strengths of community and health facilities based interventions in improving women and adolescents’ care seeking behaviors as approaches for reducing maternal mortality and improving birth outcome among low income communities of Egypt. BMC Health Serv Res. 2020;20(1):592. doi: 10.1186/s12913-020-05412-1 32600377 PMC7322855

[57] Rosales A, Sulistyo S, Miko O, Hairani LK, Ilyana M, Thomas J, et al. Recognition of and care-seeking for maternal and newborn complications in Jayawijaya district, Papua province, Indonesia: a qualitative study. J Health Popul Nutr. 2017;36(Suppl 1):44. doi: 10.1186/s41043-017-0122-0 29297380 PMC5764054

